# Testing the Effectiveness of Computerized Cognitive Training on an At-Risk Student Population

**DOI:** 10.3390/bs14080711

**Published:** 2024-08-14

**Authors:** Eugene H. Wong, Kevin P. Rosales, Lisa Looney

**Affiliations:** Department of Child Development, California State University, San Bernardino, CA 92407, USA; ewong@csusb.edu (E.H.W.); lisa.looney@csusb.edu (L.L.)

**Keywords:** computerized cognitive training, working memory, processing speed, task switching, school setting, near transfer, far transfer

## Abstract

Core constructs such as working memory, task switching, and processing speed in cognitive psychology research have prominent predictive roles in K12 students’ academic performance. Specifically, considerable empirical work shows that variability in such capabilities is linked to differences in numerous academic outcomes. Moreover, there is an increasing awareness and acceptance of the malleability of cognitive abilities. Thus, an emerging strand of research focuses on the use of computerized cognitive training to improve cognitive skills. This project addresses this issue with high-risk students attending community day schools. An in-school cognitive training program implemented (for 30 min per day) at each school site resulted in improvements for working memory, task switching, and processing speed after six total hours of participation. The current results provide evidence for the changeability of what were once thought to be static skills. Equally important, this study highlights the effectiveness of computerized cognitive training and critically extends intervention-based work to a student group that has received little attention. Implications of this work for cognitive research and educational support programs are discussed.

## 1. Individual Differences in Cognitive Abilities

Working memory (WM) is the ability to process and store information while engaging in complex tasks [1]. WM has been linked to a host of cognitive abilities such as processing speed (PS; the speed at which individuals process information) and task switching (TS; the ability to flexibly adapt between different mental sets). Moreover, WM has been linked to numerous academic outcomes such as reading comprehension [2], classroom achievement [3], and mathematics performance [4]. Given WM’s ubiquitous impact, it has become a central component within theories of cognition [5], as have the cognitive abilities to which WM has been linked (i.e., PS and TS) [6]. Researchers examining cognitive processes have consistently noted variability in WM, PS, and TS, and this variability has frequently been linked to important outcomes (e.g., reading comprehension and mathematics performance) [2,4].

Variability in cognitive processing has become a critical area of study, as it is this variability that has been used to understand differences in individuals’ performances on various tasks as well as to understand how the variability relates to other cognitive abilities. Varying levels of WM, for instance, have led to differences across an array of cognitive skills, including fluid intelligence [6], general intelligence [7], executive function [8,9], attention [10], and goal maintenance [11]. For example, differences have been found between high and low WM individuals’ performance on incongruent trials of the Stroop task (a cognitive task that requires one to process the font color of a word when at times the font color does not match the semantic meaning of the word; e.g., the word “red” presented in “blue” ink), with high WM individuals outperforming their low WM counterparts [11].

Not only do individual differences in WM relate to other cognitive abilities, but WM also contributes to variability in academic-oriented outcomes [12,13,14], with deficits in WM associated with a long list of adverse outcomes. Alloway [12] showed that impoverished WM abilities lead to difficulties with reading achievement. Specifically, children who scored low on tasks of WM had a difficult time staying up to par with their same-grade-level peers regarding reading. Moreover, writing ability seems to be negatively impacted if one has poor WM capacity, as Hoskyn and Swanson [15] found that producing quality writing (conceptual and mechanical) was largely influenced by WM capacity.

Like WM, individual differences in PS predict various academic outcomes. Within the academic context, Geary et al. [16] found that PS is a significant predictor of math achievement. Math in the classroom often occurs in a fashion where children are required to solve both simple and complex problems with time demands. Geary et al. [16] discussed that it is in time-constrained situations where PS becomes critical. From an individual differences perspective, those with higher levels of PS were more proficient on solving timed math problems with a higher success rate (i.e., greater accuracy) than those with lower PS, whose math performance was less accurate [16]. Moreover, individual differences in PS can also present both strengths and challenges in reading comprehension depending on which side of the performance continuum one lies. At the higher end, Castles et al. [17] found that PS was beneficial for reading fluency and comprehension. Specifically, individuals with a greater capacity to process information read faster and more accurately (i.e., were more fluent), which in turn led to better reading comprehension. Conversely, those with limited PS abilities suffered from low reading fluency and low reading comprehension. Lastly, writing ability is also impacted by PS. Peng et al. [18] found that PS influenced the ability to generate writing ideas, organize ideas for writing, and the ability to produce coherent writing in a timely manner. Taken together, PS is an important cognitive skill that has a notable presence in academic outcomes.

TS is a cognitive ability that holds an important place in cognitive theories of human thought [19], but also in more applied research examining its influence on academic outcomes. TS is an executive function which is important for alternating between different mental sets or rules during ongoing complex behavior [20]. Associated with this process is the ability to engage in other processes such as regulating attention [21], resisting distracting information [22], and goal maintenance [11]. TS is often an important component of measuring intelligent behavior [23], since intelligence measures that involve skills such as problem solving and reasoning depend on TS as they require one to flexibly adapt knowledge across problems.

Not surprisingly, TS impacts individuals’ performance in the academic sphere. Effective academic performance hinges on a student’s ability to navigate the dynamic and often multifaceted demands of the learning environment. TS emerges as a critical factor in this context. Functional neuroimaging studies have revealed a link between TS and activation in the dorsolateral prefrontal cortex (DLPFC) [24], a brain region that is heavily implicated in executive functions and academic performance [19]. This mental flexibility allows students to seamlessly transition between activities such as lectures and note-taking [25], or fluently switch attention between problems requiring distinct mathematical operations [26]. By facilitating the management of competing cognitive demands and promoting focus during context switches, TS skills become a cornerstone of successful academic performance.

## 2. The Malleability of Cognitive Abilities via Computerized Cognitive Training

Historically, it was a given in the field of cognition to think of cognitive abilities as static [27]. While decades of developmental research have shown growth in the complexity of cognitive abilities from infancy to adulthood (for a review see [28]), the cognitive abilities themselves were thought to be stagnant. In the last several years the field has moved towards a distinct zeitgeist; a zeitgeist where there is a strong emphasis on the malleability of cognitive abilities. This movement has led to a plethora of research studies testing the trainability of cognitive skills. In a pioneering paper by Klingberg et al. [29], WM was shown to be improved via computerized cognitive training (CCT). In a rigorously designed randomized controlled trial, Klingberg et al. [29] investigated the potential for computerized training to enhance WM in children diagnosed with Attention-Deficit/Hyperactivity Disorder (ADHD), finding that WM performance for the training group improved significantly on the trained tasks and an untrained visuospatial WM task. Notably, this improvement in cognitive function was accompanied by a reduction in parent-reported inattention and hyperactivity/impulsivity symptoms. These findings were among the first to suggest that computerized WM training is an efficacious method for improving WM among children who displayed neurodevelopmental disorders such as ADHD. What has followed is a substantial body of literature that documents the malleability of cognitive abilities via CCT (see [30]).

Additionally, other work has reported findings that CCT can improve other EFs such as TS. Zelazo et al. [31] reported that young children, young adults, and older adults benefitted from training on a TS task. Improvements in TS were found as well as improvements in abilities that were not directly trained (e.g., working memory and fluid intelligence). While there is limited research testing the effects of CCT on other abilities such as TS, these initial results nevertheless provide support that CCT can be a robust vehicle for producing boosts in a wide array of cognitive abilities.

## 3. Computerized Cognitive Training in the School Setting

As increasing emerging evidence shows that cognitive abilities are trainable (via CCT) is coupled with the long-standing acknowledgement that certain cognitive abilities (e.g., WM, PS, and TS) are essential to academic performance, there is a growing focus on CCT’s applicability in contexts outside of the lab, particularly the school setting [32,33,34]. Rabiner et al. [35] was among the first studies to test a CCT program in the school setting. In their study, first graders completed a month of cognitive training with measures of attention administered before and after the intervention. Their results showed that attentional abilities improved from pre- to post-training. These findings were corroborated by teachers’ ratings of reduced ADHD symptomology at post-test. Rabiner and colleagues [35] paved the way for a now-growing body of work showing that CCT can lead to enhancements of cognitive abilities in the school setting. In another study by Wiest et al. [32], students with learning disabilities engaged in 20 total hours of training as part of their school day. A control group engaged in an out-of-class reading activity. Wiest and colleagues [32] reported that those who received CCT improved on a measure of auditory WM. Critically, the control group who engaged in an out-of-class reading activity did not show improvements on any of the measures of cognition.

While studies have documented positive changes in cognitive abilities as a function of CCT that is implemented in the school setting, other studies have extended this work by testing whether longer training durations can impact not one cognitive ability, but multiple cognitive abilities. In a recent study by Wong et al. [33], CCT was implemented on a sample of students who attended a school that serves children with learning differences. Importantly, a hallmark of this study (in comparison to others) was that a longer training duration of 12 h was compared to a shorter training duration of six hours. The results indicated that a longer training duration led to greater improvements on WM, TS, and PS compared to the shorter training duration. These findings also showed that other abilities (such as attention) beyond WM were effectively improved in the school setting.

Similarly, in a recent study, Looney et al. [34] examined the educational implications that CCT can have when it is implemented in the school setting among children with learning differences. Like Wong et al. [33], Looney and colleagues tested the effectiveness of CCT on cognitive abilities (i.e., WM, PS, and cognitive flexibility). These abilities improved after six hours of training. These results further corroborate that CCT is a viable tool through which positive changes in cognitive abilities are possible. Moreover, there are important educational implications that can be drawn from these findings. Looney et al. [34] discussed how implementing CCT in schools as part of the school curriculum increases accessibility. That is, children who may possess cognitive hiccups, yet cannot qualify for formalized services or cannot access services external to the school environment, can benefit from the effects of CCT.

Overall, while there is a body of literature amplifying the idea that CCT is a viable intervention that can be incorporated into the school day to improve cognitive abilities, it is unknown whether CCT is effective in improving cognitive abilities in other vulnerable student bodies that are traditionally under-represented in research studies. One largely unexplored student population comprises students who typically have struggled in school as a function of a combination of factors (e.g., behavior challenges, emotional disturbances, dysfunctional family structures, and cognitive deficiencies). Struggles of this nature often lead to these students transitioning from public mainstream schools into community day schools that serve the purpose of helping these students to continue their education and graduate from high school. Testing the effectiveness of CCT across diverse student samples such as these is a necessary endeavor as it establishes the robustness of the CCT intervention and speaks to its ecological validity.

## 4. Current Study

Given the importance of specific cognitive abilities (i.e., WM, PS, and TS) on academic outcomes and the established improvements of these cognitive abilities in some student populations (via cognitive training), the current study aimed to test the effectiveness of two CCT programs for improving cognitive abilities in the school setting among at-risk youth who attend community day schools (a population less studied in the CCT literature). Specifically, we tested whether 6 h of CCT (administered during the school day) can improve WM, TS, and PS. We predicted that students would show improvements from pre- to post-training on measures of WM, TS, and PS as a function of CCT. In testing these predictions we can extend the existing literature by providing important insight as to whether CCT is a potent vehicle for producing positive changes in at-risk youth who may see restricted academic success that is driven by poor cognitive functioning. CCT can serve as a catalyst for enhancing such cognitive abilities, that in turn can bolster academic success and engagement.

## 5. Method

### Participants

This study included a sample of 62 participants (71.3% males and 28.7% females) from community day schools in Southern California who were placed at their respective school sites by the school district. These school sites are mandated by the California Department of Education and run by local school districts. Each day school serves troubled and high-risk students who have been expelled from their neighborhood school for attendance and/or behavioral concerns (e.g., school truancy and on-campus behavioral misconduct); frequently, these students are referred (to a day school) by the School Attendance Review Board (SARB) or by probation officials in the judicial system. In addition to a challenging academic curriculum, day schools focus on developing prosocial skills, resilience, and self-esteem within a setting that offers low student-to-teacher ratios. The mean age of the participants was 15.92 years (SD = 1.48). One percent of the participants identified as Asian, 15.8% identified as Black/African American, 61.4% identified as Hispanic or Latino, and 3% identified as White/Caucasian. The remainder of the participants did not identify their ethnic identity.

## 6. Materials

Participants’ cognitive abilities were evaluated with a battery of norm-referenced measures administered prior to and following training. These measures are detailed below.

*The Wechsler Intelligence Scales*. Depending upon the participant’s age, subtests from the Wechsler Intelligence Scale for Children—V (WISC-V) (Wechsler [36]) or Wechsler Adult Intelligence Test—IV (WAIS-IV) (Wechsler [37]) were given; students aged 15 years and younger were administered the WISC-V while students aged 16 years and above were given the WAIS-IV. The Coding and Symbol Search tasks (measures of PS) were administered from the age-appropriate Wechsler scale. In the Coding task, a participant is shown a key (at the top of a page) that pairs a simple shape with each number from one to nine. The individual is then asked to draw the correct shape for each presented number, working as quickly as they can from left to right, and top to bottom. The raw score for Coding is the number of correct shapes drawn in 120 s; the raw score is transformed into a scaled score (to describe processing speed) with a range of 1–19 and an average of 10. In each Symbol Search item, a participant scans a group of shapes as quickly as possible to determine if one of two target shapes is present. Ten items are presented per page and the individual works from the top to the bottom of the page. The number of correct identifications in 120 s is the raw score; this value is transformed into a scaled score with a range of 1–19 and an average of 10 to indicate processing speed.

*Delis–Kaplan Executive Function System* (DKEFS). The DKEFS (Delis et al. [38]) is a norm-referenced measure of executive function that is frequently employed in psychoeducational and neuropsychological evaluations. From the DKEFS, the Trail Making task was utilized as a measure of task switching. Trail Making is a five-part task with the first three conditions serving as primers for the fourth condition which is the actual task-switching activity. In Condition 4, a participant is asked to connect (as quickly as possible) numbers and letters in an alternating fashion (i.e., 1-A-2-B-3-C-4-D etc.). Time to completion is used as an index of task switching capability. Performance is described in the form of a scaled score with a range of 1–19 and an average of 10.

*Wide Range Assessment of Memory and Learning 3* (WRAML-3). The WRAML-3 (Adams & Sheslow [39]) is a norm-referenced measure that provides indices of verbal and visual memory as well as an index of attention/concentration. The Verbal Working Memory subtest was utilized in this project. This two-part task initially requires an individual to listen to a list of animals and non-animals; then they are asked to recall the animals first (from smallest to largest), followed by recalling the non-animal items in any order. The second part of the task requires listening to a list of animals and non-animals followed by recalling the animals first (from smallest to largest), then the non-animals (from smallest to largest). Performance on the working memory task is described with a scaled score ranging from 1–19 with an average of 10.

Computerized cognitive training was provided via two proprietary tablet-based programs developed by the University of California at Riverside Brain Game Center. These interactive activities provide cognitive training in a gamified format. The training experiences are described below.

*Recollect the Study and Sightsee*. Two CCT programs were utilized in this project. Recollect the Study is primarily a working memory task utilizing a n-back paradigm (traditionally seen in cognitive research); in the task, a participant “navigates” through space by picking up “gems” following a specific rule (e.g., pick up a gem if it matches a gem one back; pick up a gem if it matches a gem two back). Additionally, Recollect includes a memory span activity in which the individual recalls the order of a series of shapes that was just visually presented to them. Both the n-back and item-span tasks are adaptive; that is, the level of challenge is adjusted in real time according to the player’s current performance. Sightsee is a visual processing activity that requires the participant to identify targets presented on an opaque gray background as quickly as possible. As the task progresses, non-targets are introduced among the targets; thus, the adolescent must inhibit responding as well as identifying correct targets. As with Recollect, Sightsee is adaptive; thus, an optimal level of challenge is continuously provided.

## 7. Procedure

Following Institutional Review Board approval, administrative staff at each school site were informed of this project and its objectives. Subsequently, at each school, students were told about the project in their respective classroom by research personnel. All students at a day-school site were invited to participate. Interested students were asked to secure informed consent from a parent (or guardian) if they were a minor, then to provide assent themselves. If the potential participant was 18 years or older, they provided informed consent.

Prior to beginning cognitive training, each participant’s WM, TS, and PS were evaluated in order to establish a baseline for each individual. Subsequently, participants were randomly assigned to one of the two training programs (i.e., Recollect and Sightsee). They, then completed CCT for 30 min daily, Monday through Thursday, in a small group setting with one research assistant; the student-to-research assistant ratio was no larger than 3:1 in order to ensure that each participant had adequate support in learning the training game and while engaged with the task. All research assistants were extensively trained on the games prior to interacting with the adolescents. Participants were pulled from their classroom to complete the daily training in a quiet room on the school campus. All training sessions were integrated into the school day to maximize the likelihood that trained skills would transfer to the students’ typical school-day classroom activities. After six hours of training was attained, the measures of WM, TS, and PS were re-administered (to provide a post-training indicator of these cognitive abilities). Participants received snacks daily for their participation; additionally, for each day of participation, participants’ names were included in a monthly draw for a gift card to a local student-selected eatery.

## 8. Design and Statistical Analysis

The current study implemented a 2 × 2 mixed-factorial design (time: pretest, post-test × game condition: Recollect, Sightsee). Time of testing was the within-subject factor while the game condition was the between-subject factor. Measures of WM, TS, and PS were utilized as the dependent variables.

A 2 × 2 mixed factorial ANOVA was implemented to analyze the data from this study. The dependent variables were standardized scores on measures of WM, TS, and PS. Partial eta-squared was used as the effect size where a small effect was indicated by values ranging from 0.01 to 0.05, a medium effect was denoted by values ranging from 0.06 to 0.13, and a large effect was indicated by values of 0.14 and above.

## 9. Results

The results for 62 participants (Recollect, *N* = 32; Sightsee, *N* = 30) are reported here. No outliers were found in the current data set. Assumptions of normality and homogeneity of variance were met. Results for WM, TS, and PS are discussed below.

### 9.1. Working Memory

**Figure 1:**
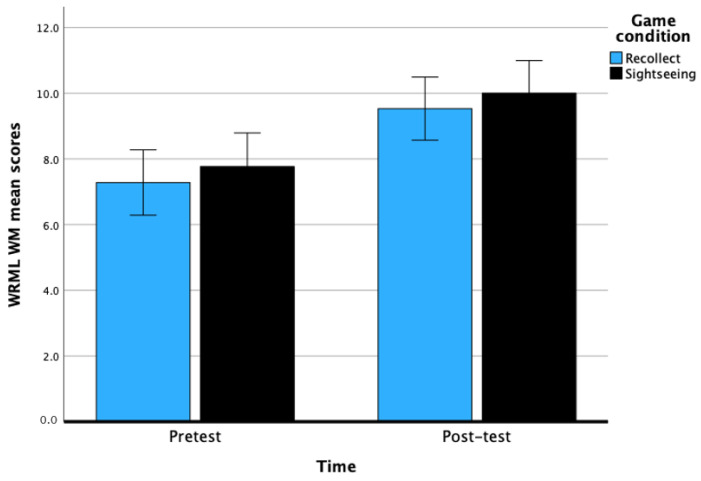
Pretest to post-test differences of WM as function of game condition after 6 h of training. Bars represent the 95% CI.

There was a significant main effect of time on WM, *F*(1, 60) = 54.70, *p* < 0.001, with a large effect, η^2^ = 0.48. There was a significant improvement from pretest (M = 7.52, *SE* = 0.36) to post-test (M = 9.77, *SE* = 0.35) scores for WM. The main effect of game condition was not significant, *F*(1, 60) = 0.56, *p* = 0.46, with a small effect size of η^2^ = 0.01. The interaction between time and game condition was not significant, *F*(1, 60) = 0.001, *p* = 0.98. Overall, this set of results indicate that 6 h of CCT leads to significant improvement in WM among the day-school students. See Figure 1 below.

### 9.2. Task Switching

**Figure 2:**
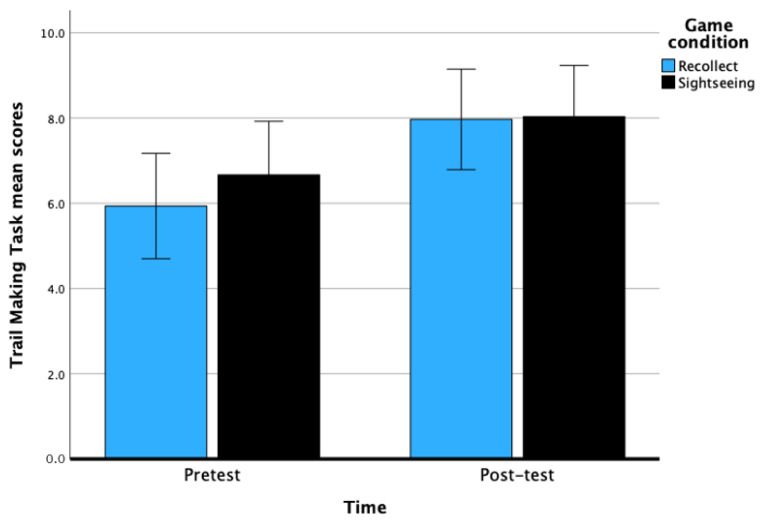
Pretest and post-test scores of TS across game conditions after 6 h of CCT. Bars represent the 95% CI.

There was a significant main effect of time on TS, *F*(1, 60) = 17.26, *p* < 0.001, with a large effect, η^2^ = 0.23. There was a significant improvement from pretest (M = 6.30, *SE* = 0.44) to post-test (M = 8.00, *SE* = 0.42) scores for TS. The main effect of game condition was not significant, *F*(1, 59) = 0.28, *p* = 0.60, with a small effect size of η^2^ = 0.01. The interaction between time and game condition was not significant, *F*(1, 60) = 0.66, *p* = 0.42. Overall, this set of results indicate that 6 h of CCT leads to significant improvement in TS among the day-school students. See Figure 2 below.

## 10. Processing Speed

### 10.1. Coding

**Figure 3:**
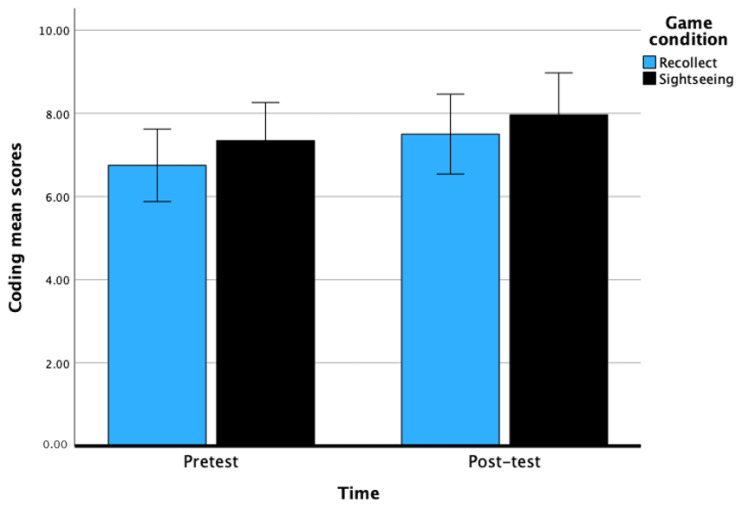
Pretest and post-test scores of Coding across game conditions after 6 h of CCT. Bars represent the 95% CI.

There was a significant main effect of time on Coding, *F*(1, 59) = 8.65, *p* < 0.01, with a medium effect, η^2^ = 0.13. There was a significant improvement from pretest (M = 7.04, *SE* = 0.32) to post-test (M = 7.73, *SE* = 0.35) scores for Coding. The main effect of game condition was not significant, *F*(1, 59) = 0.72, *p* = 0.40, with a small effect size of η^2^ = 0.01. The interaction between time and game condition was not significant, *F*(1, 60) = 0.08, *p* = 0.78. Overall, this set of results indicate that 6 h of CCT leads to significant improvement in PS among the day-school students. See Figure 3 below.

### 10.2. Symbol Search

**Figure 4:**
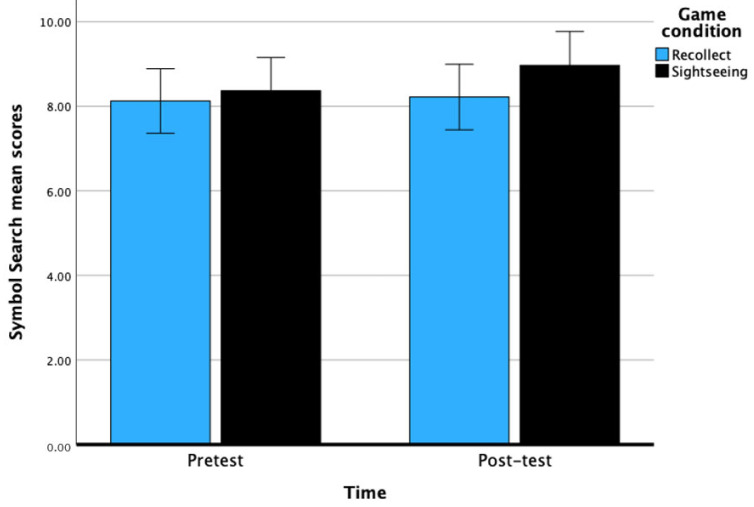
Pretest and post-test scores of Symbol Search across game conditions after 6 h of CCT. Bars represent the 95% CI.

There was no main effect of time on Symbol Search scores, *F*(1, 60) = 2.05, *p* = 0.16, with a small effect, η^2^ = 0.03. Specifically, there was no significant improvement from pretest (M = 8.24, *SE* = 0.27) to post-test (M = 8.59, *SE* = 0.28) scores for Symbol Search. The main effect of game condition was not significant, *F*(1, 60) = 0.99, *p* = 0.32, with a small effect size of η^2^ = 0.02. The interaction between time and game condition was not significant, *F*(1, 60) = 1.09, *p* = 0.30. Overall, this set of results indicate that for this measure of PS, 6 h of CCT did not lead to improvements in Symbol Search scores. See Figure 4 below.

## 11. Discussion

The primary purpose of this project was to examine the effectiveness of CCT in improving cognitive abilities among a high-risk student population (attending community day schools) that has received little attention in the empirical literature. While an evolving body of work (see [32,33,34]) documents the effectiveness of CCT in building cognitive skills such as WM, TS, and PS among various student populations, the focus of the current project is unique. As a group, community day-school students represent adolescents with significant academic challenges, as well as social-emotional, mental health, and often legal concerns that negatively impact their school experience. As a result, these students often experience low academic performance, are not engaged with school, and experience negative future trajectories (e.g., not graduating from school, not being able to sustain employment, etc.) that clearly impact their adult lives.

The current results suggest that critical cognitive skills that are predictive of positive academic outcomes can be enhanced after six hours of CCT. Specifically, three out of the four total effects were significant (WM, TS, and Coding: a measure of PS). This set of findings highlights the potency and validity of CCT as an intervention by demonstrating that it can positively impact a host of cognitive abilities. Importantly, the CCT yielded moderate-to-large effect sizes which are indicative of the practical significance of this intervention. Given the central nature of WM, TS, and PS in both prominent cognitive theoretical models as well as applied educational research, our results offer evidence that community day-school students’ school experience can be effectively supported with CCT.

### 11.1. The Centrality of WM, TS, and PS

Cognitive models that have sought to explain the complex dynamics characterizing human cognitive processes have invariably addressed constructs such as WM, TS, and PS [19]. These models and associated research highlight the complex relations among these cognitive abilities, the associations between these abilities and educational outcomes, and the presence of individual differences in these capabilities that explain performance variability among individuals (see [40]). Because of the importance of WM, TS, and PS, they are invariably examined in cognitive and educational research.

The results of the current project provide further evidence for a continued empirical focus on these cognitive capabilities for a number of reasons. First, our results illustrate the malleability of WM, TS, and PS in an adolescent population. Consistent with studies finding that cognitive abilities are malleable [29,32,33,34], it appears that cognitive abilities are not static, but instead can be “grown”, much as muscles may be enhanced by physical training. Such findings have important implications as they suggest that cognitive skills may not have a hard “ceiling” (i.e., a maximal level of development) that is determined early in life. Future cognitive research should seek to better understand the developmental course of specific cognitive abilities. Particular attention should be given to identifying how malleable the different cognitive skills are, whether developmental improvement plateaus, and how cognitive abilities may be increasingly integrated (connected) with one another via CCT. Addressing such issues will provide a more comprehensive understanding of human cognitive abilities, while simultaneously contributing to an evolving and fuller understanding of educational supports that can benefit K12 students.

### 11.2. Integrating CCT into the School-Day

During the past 20 years, a body of research has examined the effectiveness of CCT for improving cognitive abilities [30]. While some early work questioned whether CCT was viable as a targeted intervention for cognitive skills such as WM, there is now an increasing consensus that cognitive training leads to near-transfer effects (see [41]). What is less clear in existing research is whether there is far transfer of training; that is, does training a specific skill (e.g., WM) lead to improvement in other outcomes (e.g., reading achievement) that were not directly singled out for training?

As the effectiveness of CCT has been increasingly documented, a central question regarding the context of training has emerged. That is, does the setting in which training occurs matter? Historically, while much CCT research has been connected to educational outcomes (i.e., cognitive training was used to improve cognitive skills that are important to school), the training has not occurred in the school. Instead, CCT was typically offered within a clinic setting or was completed in the participant’s home (e.g., [29]). More recently, several studies have successfully integrated CCT into the school day with very positive results [32,33,34]. These studies show that cognitive training can be included into the school-day curriculum without interfering with typical school operations. The benefit of such an approach is that students receive training in the same setting in which they will employ the trained cognitive skills. The results of the current project add to this line of research and importantly indicate that CCT can be effectively integrated in a less traditional (i.e., day school) academic setting.

### 11.3. Limitations

While we support the effectiveness of CCT for improving cognitive abilities in the school setting, there are a few limitations worth highlighting. First, there is a lack of a “true” control group in this project as the school district we partnered with required that all participants receive training. In the future, it would be valuable to create a control group by implementing staggered training during a school year. That is, an initial group of students receives training while there is second group of participants who has contact with the program but does not begin training until a later point in the academic year. With such an approach, all students will still receive CCT, but we can more clearly evaluate the effectiveness of training. Second, we administered only one measure of WM and TS, which hinders construct validity. More measures of each construct should be tested in this vein of work in the future. Finally, while our sample size was not concerningly small, this work could benefit from larger sample sizes to solidify the effectiveness of CCT.

### 11.4. Future Directions

The promising findings from recent school-day CCT programs should provide the impetus for work that addresses a number of critical questions in this area of research. First, as there is increasingly consistent evidence for the effectiveness of CCT, it is important to better understand the mechanisms of change (i.e., how does improvement occur?). For example, are there structural changes in the brain (following training) that may be identified via imaging studies? Or, from a cognitive psychology perspective, is there a modification in the underlying structure of cognitive abilities that explains the outcomes reported by researchers? Delving into such (and other related) questions will provide important insights at a basic knowledge level as well as at the intervention level so that CCT can be as individualized as possible.

Second, there is a need to understand how best to support students who are engaged in training. That is, it is not feasible to assume that students can simply be given a device and instructed to engage with the CCT activities. The dynamics that evolve between a trainer and a trainee mostly likely contribute to the effectiveness of cognitive training. In fact, some may argue that this is a critical factor in whether any improvement in cognitive abilities is demonstrated following training. Work in this area will require sophisticated mixed methods designs, but it will ultimately prove to be highly valuable in building our understanding of cognitive abilities and CCT.

Finally, it is important to document the sustainability of the effects of CCT. While it is meaningful to demonstrate change in cognitive abilities immediately following cognitive training, it is especially important to show evidence that the change is sustained. Longitudinal work embedded into the school day that can follow a cohort of students will provide important and deeper understanding of cognitive abilities for cognitive psychologists and practitioners in educational settings.

## 12. Conclusions

The current project extends the CCT research literature in several notable ways. First, we provide evidence of the effectiveness of CCT implemented during the school day. Increasingly, it is clear that cognitive training has validity as an intervention. Second, this project provides initial findings which suggest that high-risk students benefit from such interventions (when it is integrated into their regular school-day experiences). This result is important as this traditionally hard-to-reach group is likely in need of a range of educational supports in order to be academically successful. Finally, the current results highlight the malleability of cognitive abilities; this conclusion has important implications for cognitive psychology in which there is an evolving contemporary zeitgeist that views cognitive abilities as dynamic versus static.

## Data Availability

The data presented in this study are available on request.
